# *Forsythia suspensa* Leaves Triterpenoids Induce Breast Cancer Cell Apoptosis via the Mitochondrial Pathway

**DOI:** 10.1002/fsn3.70664

**Published:** 2025-08-03

**Authors:** Xiao Li, Xuefang Wang, Ling Chen, Erjuan Ning, Hang Fan, Jun Liang, Xinxin Lai, Lulu Zhang, Yingkui Gao, Yan Li, Panpeng Wei, Liqin Yu, Yi Fan, Xuebing Wang

**Affiliations:** ^1^ Henan Province Engineering Research Center of Natural Medicine Quality Control and Production Process Zhengzhou University of Industrial Technology Zhengzhou China; ^2^ Henan High Tech Industry Co., Ltd. Henan Academy of Sciences Zhengzhou China; ^3^ Henan Natural Products Biotechnology Co., Ltd. Henan Academy of Sciences Zhengzhou China; ^4^ College of Veterinary Medicine Henan Agricultural University Zhengzhou China; ^5^ Zhongyuan Chuangke (Henan) Biotechnology Co., Ltd. Luoyang China

**Keywords:** apoptosis, breast cancer cell, *Forsythia suspensa* leaves, triterpenoids mitochondrion

## Abstract

*Forsythia suspensa* is a traditional Chinese medicine. The leaves of *F. suspensa* , specifically the dried leaves of the plant, are commonly consumed as tea in China. In this study, *F. suspensa* leaves triterpenoids (FLT) were isolated and purified from the dried leaves of *F. suspensa* , and their in vitro antitumor activity as well as associated molecular mechanisms were systematically investigated. First, the primary components of FLT were determined using high‐performance liquid chromatography (HPLC), and the results revealed ursolic acid (70.67%), oleanolic acid (16.23%), and betulinic acid (4.59%) as the major components. Next, the results of the 3‐(4,5‐dimethylthiazol‐2‐yl)‐2,5‐diphenyltetrazolium bromide (MTT) assay showed that FLT exhibited strong antiproliferative activity (*p* < 0.05) toward human breast cancer MCF‐7 and MDA‐MB‐231 cells. Moreover, the flow cytometry detection of apoptosis using Annexin V‐fluorescein isothiocyanate/propidium iodide (Annexin V‐FITC/PI) revealed that FLT significantly induced apoptosis in MCF‐7 and MDA‐MB‐231 cells in a dose‐dependent manner (*p* < 0.001). Laser confocal microscopy showed that FLT was mainly located in the mitochondria and lysosome of cells. Meanwhile, after the FLT (15 μg/mL) treatment of MCF‐7 and MDA‐MB‐231 cells, the mitochondrial membrane potential (MMP) (*p* < 0.001) and intracellular reactive oxygen species (ROS) level (*p* < 0.001) were significantly reduced. Finally, western blotting demonstrated that FLT (10 and 15 μg/mL) significantly reduced B‐cell lymphoma‐2 (Bcl‐2) (*p* < 0.001) and cysteinyl aspartate specific proteinase‐3 (caspase‐3) protein levels (*p* < 0.001), but significantly increased Bcl‐2 homologous antagonist/killer (Bak) protein (*p* < 0.05 or *p* < 0.001), cleaved‐caspase‐3 protein (*p* < 0.001), dynamic‐related protein 1 (DRP1), and mitochondrial fission 1 protein (FIS1) levels (*p* < 0.01 or *p* < 0.001). Taken together, the results indicated that FLT promoted the apoptosis of MCF‐7 and MDA‐MB‐231 cells by activating the mitochondrial pathway. This effect may be attributed to FLT affecting the expression of the Bcl‐2 family in mitochondria by promoting DRP1 and FIS1‐mediated mitochondrial division, thereby activating the cleavage of caspase‐3 and ultimately leading to cell apoptosis.

## Graphical abstract

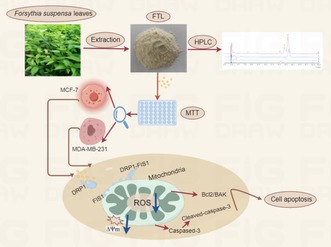

**Abbreviations**
Annexin V‐FITC/PI: Annexin V‐fluorescein isothiocyanate/propidine iodide
BA: betulinic acid
Bak: Bcl‐2 homologous antagonist/killer
BCA: bicinchoninic acid
Bcl‐2: B‐cell lymphoma‐2
Bcl‐W: BCL‐2 like 2
Bcl‐XL: B‐cell lymphoma‐extra large
Caspase‐3: cysteinyl aspartate‐specific proteinase‐3
DCFH‐DA: 2′,7′‐dichlorodihydrofluorescein diacetate
DMSO: dimethyl sulfoxide
DRP1: dynamic‐related protein 1
ECL: enhanced chemiluminescence
ER: endoplasmic reticulum
FBS: fetal bovine serum
FIS1: mitochondrial fission 1 protein
FLT:
*Forsythia suspensa* leaves triterpenoids
HPLC: high‐performance liquid chromatography
IC_50_
: half maximal inhibitory concentration
MCL‐1: myeloid cell leukemia 1 protein
MMP: mitochondrial membrane potential
MTT: 3‐(4,5‐dimethylthiazol‐2‐yl)‐2,5‐diphenyltetrazolium bromide
OA: oleanolic acid
PBS: phosphate‐buffered saline
PVDF: polyvinylidene difluoride
Rh123: rhodamine‐123
RIPA: radioimmunoprecipitation assay
ROS: reactive oxygen species
RPMI 1640: Roswell Park Memorial Institute 1640
SDS‐PAGE: sodium dodecyl sulfate polyacrylamide gel electrophoresis
TBST: tris‐buffered saline and tween
UA: ursolic acid
UV: ultraviolet‐visible spectroscopy

## 1 Introduction

Breast cancer is the most frequent cancer in women (both cases and deaths), and its incidence has been increasing yearly (11.6% of all cancers globally) (Bray et al. 2024). It poses a great threat to women's health. At present, the major treatment modalities for breast cancer include mastectomy, chemotherapy, radiotherapy, and combination therapy (Gray and Campbell 2018). However, these methods have severe side effects such as toxicity to healthy cells and immune damage (Tariq et al. 2015). Therefore, there is an urgent need for developing new therapeutic methods for breast cancer.

More than 50% of all the drugs in modern therapeutics are based on natural products and their derivatives, including various phytochemicals from herbal medicines (Pan et al. 2013; Schmidt et al. 2008; Chang et al. 2024). Numerous studies have demonstrated that plant triterpenoids, polyphenolic compounds, and other phytochemicals can effectively inhibit the progression of breast cancer invasion and metastasis, exhibiting significant potential in the treatment and suppression of breast cancer (Aly et al. 2024; El‐Nashar et al. 2022). The extract of *Dolomiaea costus*, primarily composed of polyphenolic compounds, has shown notable inhibitory effects on the proliferation of breast cancer MCF‐7 cells (El‐Nashar et al. 2023). Studies have shown that dietary intervention may affect the availability of nutrients in tumors, thereby improving the effect of cancer treatment (Xiao et al. 2024; Yuan et al. 2020; Sun‐waterhouse et al. 2024; Cong 2024). Consuming green tea may help women prevent breast cancer. After surgery and chemotherapy, the intake of green tea may reduce the possibility of recurrence and metastasis in patients with breast cancer (Santos et al. 2023; Marín et al. 2023). El‐Nashar et al. (2024) showed that the combination of cisplatin and white pitaya fruit extract has a significant inhibitory effect on HeLa cell apoptosis. Meanwhile, owing to the presence of bioactive ingredients, plant‐based food may play an inhibitory role in the promotion and development of cancer by scavenging free radicals, repairing DNA damage, inhibiting cell proliferation, inducing apoptosis, exhibiting antioxidant and anti‐inflammatory effects, and regulating immunity (Liu 2013; Xia et al. 2023; Yin et al. 2021).

According to the records of “Chinese Materia Medica,” the stems and leaves of *Forsythia suspensa* (Thunb.) Vahl of the Oleaceae family have a bitter taste and cold nature and can be used to treat cardiopulmonary heat accumulation (National Administration of Traditional Chinese Medicine 1999). In Shanxi, Henan, Hebei, and other provinces of China, the young leaves of *F. suspensa* are often consumed as health tea. In 2017, *F. suspensa* leaves were approved as food raw materials (Chai et al. 2022). The leaves mainly contain phenylethanoid glycosides, lignans, flavonoids, triterpenoids, and other chemical components and exhibit antibacterial, antiviral, antioxidant, liver‐protecting, blood lipid‐lowering, blood glucose‐lowering, and antitumor activities (Kang and Wang 2010; Zhang et al. 2023; Liu et al. 2024; Zhang 2020). Modern pharmacological research has shown that triterpenoid compounds such as 20(s)‐dammar‐24‐ene‐3β,20‐diol‐3β‐acetate and ambrolic acid isolated from *F. suspensa* have good inhibitory activity on gastric cancer and liver cancer cells (Shi et al. 2009). In addition, the leaves of *F. suspensa* contain abundant ursolic acid (UA), oleanolic acid (OA), and other pentacyclic triterpenoids (Wang, Chen, et al. 2023; Wang, Jiang, et al. 2023; Yuan et al. 2015). UA exhibits several biological activities, including antiviral, anti‐hepatitis, blood lipid‐lowering, and blood glucose‐lowering effects, and has a good inhibitory effect on tumor cell proliferation (Kong et al. 2013; Wang et al. 2017; Shi et al. 2016). Meanwhile, OA can inhibit the growth of many tumor cell types, such as human lung giant cell carcinoma PGCL3, human lung fibroblast WI‐38, human liver cancer (HepG2, HuH7), ovarian cancer HO‐8910 cells, and others (Fu et al. 2005; Wu et al. 2009). Therefore, triterpenoids may be the major antitumor components in *F. suspensa* leaves. At present, there is no report on the antitumor effect of *F. suspensa* leaves triterpenoids (FLT) on breast cancer cells, and its mechanism remains unclear.

Mitochondria are important organelles that produce intracellular energy and play crucial roles in signal transduction, cell proliferation, differentiation, autophagy, and cellular immunity, and regulate several programmed cell death pathways, including apoptosis (Zong et al. 2016). At the same time, mitochondria are highly dynamic organelles, and the imbalance between their division and fusion often leads to structural changes, dysfunction, cellular stress, and death. Thus, inhibiting mitochondrial metabolism may effectively prevent tumor progression (Weinberg and Chandel 2015; Wang, Chen, et al. 2023; Wang, Jiang, et al. 2023).

In the present study, FLT were isolated and purified, and their antitumor activities were investigated. A major focus of the study was to assess the effect of FLT on the apoptosis of human breast cancer cells MCF‐7 and MBA‐MB‐231 and the mechanism of inducing apoptosis through the mitochondrial pathway.

## 2 Materials and Methods

### 2.1 Chemicals and Reagents

All chromatographic reagents were purchased from Thermo Fisher Scientific (China) Co. Ltd. The D101 macroporous resin was purchased from Sunresin New Materials Co. Ltd. (Xi'an). Roswell Park Memorial Institute 1640 (RPMI 1640) medium was purchased from Gibco Life Technologies (USA), penicillin mixed solution from Beijing Solarbio Biotechnology Co. Ltd., standard fetal bovine serum (FBS) from Zhejiang Tianhang Biotechnology Co. Ltd. and trypsin from Amreseo Inc. (USA). Annexin V‐fluorescein isothiocyanate/propidine iodide (Annexin V/PI) kits, western and radio immunoprecipitation assay (RIPA) cell lysates, and bicinchoninic acid assay (BCA) protein concentration kits were bought from Beyotime Biotechnology Co. Ltd. Rhodamine‐123 (Rh123) and 2′,7′‐dichlorodihydrofluorescein diacetate (DCFH‐DA) were purchased from Sigma Corporation (USA); polyvinylidene fluoride (PVDF) films were sourced from Millipore Corporation; and cysteinyl aspartate‐specific proteinase‐3 (caspase‐3), B‐cell lymphoma‐2 (Bcl‐2), Bcl‐2 homologous antagonist/killer (Bak), and β‐Actin were commercially obtained from Santa Cruz Biotechnology.

### 2.2 Extraction and Purification of FLT

*F. suspensa* leaves were collected from Luanchuan, Henan Province, in July 2021. The leaves were authenticated as *F. suspensa* (Oleaceae) by Prof. Chengming Dong from Henan University of Chinese Medicine. The voucher specimen (No. 210701) has been deposited in the Key Laboratory of Natural Products, Henan Academy of Sciences. Approximately 5000 g of dried leaves were crushed into a coarse powder and extracted three times for 1 h each with 8 times of 95% ethanol at 80°C. The resulting ethanol extract (concentration = 95%) was evaporated at 55°C under vacuum to remove ethanol. Then, the residue was redissolved in 5000 mL of water. After filtration, the supernatant was purified via the D101 macroporous resin column (100 × 1600 mm) and eluted with 5000 mL of 70% ethanol and 3000 mL of 95% ethanol sequentially. The eluate of 95% ethanol was concentrated in a vacuum at 60°C to obtain a yellow–white powder (FLT).

### 2.3 Ultraviolet‐Visible Spectroscopy (UV) and High‐Performance Liquid Chromatography (HPLC) Analysis

The contents of total triterpenoid in FLT were determined using UV spectrophotometry. First, OA reference substance was accurately weighed to prepare control solutions at the concentrations of 40, 80, 120, 160, and 200 μg/mL with methanol. In addition, FLT powder was accurately weighed to prepare the sample solution at 100 μg/mL with methanol. Approximately 1 mL of the above solutions was added to 10 mL test tubes with stoppers. After volatilizing the solvent in an oven at 75°C, 5% vanillin–glacial acetic acid solution and 0.8 mL perchloric acid solution were added, followed by shaking. The mixture was placed in a 70°C water bath for 25 min and cooled to room temperature for 10 min. Then, 4 mL glacial acetic acid solution was added and mixed evenly. The absorbance of the solution was determined at 546 nm, and the content of total triterpenoid was expressed as the percent mass in the extract.

The contents of UA, OA, and betulinic acid (BA) in FLT were determined using LC‐20AT HPLC (diode array detector, Shimadzu Company, Japan). The following chromatographic conditions were followed: Agilent ZORBAX SB‐C18 column (4.6 × 250 mm, 5 μm); mobile phase, methanol: 0.1 mol/L ammonium acetate solution (85:15); flow rate, 1.0 mL/min; and detection wavelength, 210 nm. First, appropriate amounts of UA, OA, and BA control substances were accurately weighed to prepare their respective stock solutions containing 0.2 mg of control substance per 1 mL of methanol, which were stored in a refrigerator at 4°C for subsequent experiments. 0.04 g FLT powder was also accurately weighed in a 50 mL triangular flask. Then, 10 mL of 95% ethanol was added and ultrasound treatment was performed for 30 min, followed by cooling. After complementing the weight loss, the solution was shaken and filtered through a 0.45 μm filter membrane. The filtrate was continuously collected to determine the contents of UA, OA, and BA in FLT.

### 2.4 Cell Culture

The human breast cancer cells MCF‐7 and MDA‐MB‐231, liver cancer cells HepG2 and SNU739, colon cancer cells HT29 and HCT116, normal liver cells HL‐7702, and normal breast epithelial cells MCF‐10A were purchased from the Shanghai Institute of Cell Biology, Chinese Academy of Sciences. The cancer cells were cultured in RPMI‐1640 medium containing 10% FBS, 100 IU/mL penicillin, and 100 IU/mL streptomycin in an incubator at 37°C with 5% CO_2_, and cells in the logarithmic growth phase were used for subsequent experiments.

### 2.5 Cell Viability Assay

MCF‐7, MDA‐MB‐231, HepG2, SNU739, HT29, HCT116, HL‐7702, and MCF‐10A cells were seeded in 96‐well plates at a concentration of 5000 cells per well. After culturing for 24 h, the cells were treated with FLT at different concentrations (2.5, 5, 10, 20, and 40 μg/mL). In addition, a blank control and positive drug (mitoxantrone) control groups were set. After culturing for 48 h, 50 μL 3‐(4,5‐dimethylthiazol‐2‐yl)‐2,5‐diphenyltetrazolium bromide (MTT) reagent (1 mg/mL) was added to each well, followed by 4 h of incubation. Then, the supernatant was discarded and 100 μL dimethyl sulfoxide (DMSO) was added to each well. After the blue‐colored crystal violet dye was completely dissolved, the absorbance value was measured at a 570 nm wavelength using a microplate reader to calculate its half maximal inhibitory concentration (IC_50_) value. Each experiment was repeated at least three times.

### 2.6 Annexin V‐FITC/PI Staining

The Annexin V‐FITC/PI double staining method was employed to detect apoptosis in breast cancer cells. First, MCF‐7, MDA‐MB‐231, and HL‐7702 cells were seeded into 6‐well plates at a concentration of 5 × 10^4^ cells per well. After 24 h of culture, FLT solution at different concentrations (5, 10, and 15 μg/mL) was added and incubated for 24 h. Then, the cells were digested with trypsin and collected and washed twice with phosphate‐buffered saline (PBS). Next, 5 μL Annexin V‐FITC and 5 μL PI were added to each well and kept in the dark at room temperature for 15 min, followed by washing with PBS three times. Apoptosis was detected using flow cytometry (BD Biosciences, San Jose, CA, USA). Each experiment was repeated three times.

### 2.7 Cellular Uptake and Localization

The uptake and localization of FLT in cells were detected using laser confocal microscopy (Leica, Wetzlar, Germany). MCF‐7 and MDA‐MB‐231 cells were seeded into a culture dish at a density of 5000 cells per well and incubated for 24 h, and then treated with FLT solution at different concentrations (2.5, 5, 10, and 15 μg/mL). After a certain incubation period (10 min, 30 min, 1 h, and 2 h), the uptake of FLT by cells was observed. The cells were stained with various organelle‐specific fluorescent dyes, such as Mito‐tracker for mitochondria (Yeasen, Shanghai, China), Lyso‐tracker for lysosomes (Beyotime, Jiangsu, China), ER‐tracker for endoplasmic reticulum (ER) (Beyotime, Jiangsu, China), and Golgi‐tracker for the Golgi (Beyotime, Jiangsu, China), for 30 min and washed with PBS three times; the localization of FLT by cells was observed.

### 2.8 Mitochondrial Membrane Potential (MMP; 1ψm)

MMP was determined using the Rh123 staining method. MCF‐7 and MDA‐MB‐231 cells were seeded into 6‐well plates at a concentration of 5 × 10^4^ cells per well. After 24 h of culture, FLT solution at different concentrations (5, 10, and 15 μg/mL) was added and incubated for 24 h. Then, cells were digested using trypsin and collected and washed twice with PBS. The cells were subsequently incubated with PBS solution containing 15 μg/mL Rh123 dye for 25 min in the dark at 37°C. After washing with PBS three times, MMP was determined using flow cytometry. Each experiment was repeated three times.

### 2.9 DCFH‐DA Staining

The intracellular reactive oxygen species (ROS) level was determined via the DCFH‐DA staining method. First, MCF‐7 and MDA‐MB‐231 cells were seeded into a 6‐well plate at a concentration of 5 × 10^4^ cells per well. After 24 h of culture, FLT solution at different concentrations (5, 10, and 15 μg/mL) was added and incubated for 24 h. Then, the cells were digested with trypsin and collected. After being washed twice with PBS, the cells were incubated with PBS solution containing 10 μM DCFH‐DA for 25 min in the dark at 37°C. Following incubation and final washing with PBS three times, flow cytometry was performed for ROS level estimation. Each experiment was repeated three times.

### 2.10 Western Blotting

The effect of FLT on the level of the apoptosis‐related protein caspase‐3, cleaved‐caspase‐3, Bcl‐2, and Bak as well as of the mitochondrial division‐related proteins, dynamic‐related protein 1 (DRP1), and mitochondrial fission 1 protein (FIS1) in MDA‐MB‐231 and MCF‐7 cells was measured using western blotting. The cells were treated with FLT at the concentrations of 5, 10, and 15 μg/mL for 24 h and were harvested and centrifuged. Then, the cells were lysed with RIPA lysis buffer. The total concentrations of protein were measured using the BCA Assay Kit (Beyotime, China). The total lysates were denatured in 5 × SDS‐loading buffer at 100°C for 10 min. Equal amounts of total proteins were separated using 12% sodium dodecyl sulfate polyacrylamide gel electrophoresis (SDS‐PAGE) for 2 h and then transferred onto PVDF membranes. The membranes were blocked with 5% skimmed milk at room temperature for 2 h, washed in tris‐buffered saline and tween 20 (TBST) buffer, incubated with corresponding primary antibodies, and shaken overnight at 4°C. After being washed three times with TBST, the membranes were incubated with appropriate HRP‐conjugated secondary antibody and then washed three times with TBST. Enhanced chemiluminescence (ECL) luminescent solution was then added in the dark and the membranes were photographed using a gel imaging system. Relative protein expression was analyzed using β‐actin as an internal reference.

### 2.11 Statistical Analyses

All the data are presented as the mean ± SD and analyzed using Student's *t* test or analysis of variance (ANOVA) followed by *q*‐test.

## 3 Results

### 3.1 UA, OA, BA, and Total Triterpenoid Levels in FLT

**FIGURE 1:**
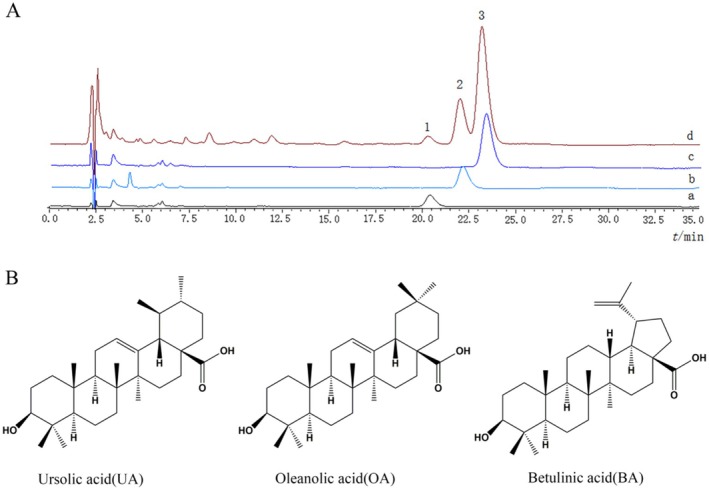
HPLC chromatogram and chemical structures. (A) [a] HPLC chromatogram of standard sample BA, [b] HPLC chromatogram of standard sample OA, [c] HPLC chromatogram of standard sample UA, [d] HPLC chromatogram of FLT (1. BA, 2. OA, 3. UA). (B) The chemical structures of UA, OA, and BA.

After extracting 5000 g of *F. suspensa* leaves with 95% ethanol, the extract was enriched and purified using the D101 macroporous resin column to produce (4.10 ± 0.74) g of yellow–white FLT powder. Colorimetry revealed that the total triterpenoid level in FLT was (95.6% ± 2.28%), whereas HPLC estimated that UA, OA, and BA levels in FLT were (70.67% ± 1.3%), (16.23% ± 2.1%), and (4.59% ± 1.5%) (Figure 1A), and their chemical structures are shown in Figure 1B.

### 3.2 Cell Viability

MTT assay was used to assess the antiproliferative activity of FLT, UA, OA, and BA on six human tumor cells and two human normal cells, including MCF‐7, MDA‐MB‐231, HepG2, SNU739, HT29, HCT116, HL‐7702, and MCF‐10A. The results showed that FLT, UA, OA, and BA all exhibited good antiproliferative activity on the tumor cells. Among the four, FLT had the highest antiproliferative effect on human breast cancer cell lines (MCF‐7 and MDA‐MB‐231), with the IC_50_ values of 6.52 ± 0.79 and 7.90 ± 0.94 μg/mL, and had a weak antiproliferative effect on human normal liver cells and breast epithelial cells (Table 1).

**TABLE 1:** IC_50_ values for the inhibition of human tumor cells of FLT (Mean ± SD, *n* = 6).

Sample	IC_50_(μg/mL)							
	MCF‐7	MDA‐MB‐231	HepG2	SNU739	HT29	HCT116	HL‐7702	MCF‐10A
FLT	6.52 ± 0.79^d^	7.90 ± 0.94^c^	10.05 ± 0.33^d^	9.08 ± 0.71^d^	20.40 ± 1.18^c^	21.85 ± 1.58^c^	57.90 ± 1.54^b^	89.62 ± 3.28^a^
UA	7.29 ± 0.45^c^	9.51 ± 0.86^b^	12.05 ± 0.54^c^	10.91 ± 0.96^c^	26.41 ± 0.40^b^	28.98 ± 1.90^b^	48.43 ± 1.32^d^	69.66 ± 2.56^d^
OA	10.27 ± 0.67^b^	13.46 ± 0.56^a^	16.34 ± 0.77^b^	14.82 ± 0.56^b^	20.43 ± 1.48^c^	21.89 ± 1.67^c^	55.65 ± 1.76^c^	77.51 ± 2.75^c^
BA	11.82 ± 0.20^a^	14.17 ± 0.47^a^	26.00 ± 1.16^a^	28.06 ± 1.34^a^	44.01 ± 1.46^a^	43.00 ± 1.79^a^	68.88 ± 1.89^a^	80.80 ± 2.58^b^
Mitoxantrone	0.55 ± 0.17^e^	1.03 ± 0.26^d^	1.65 ± 0.21^e^	4.04 ± 0.32^e^	5.13 ± 0.98^d^	5.74 ± 1.23^d^	3.20 ± 0.65^e^	2.44 ± 0.47^e^

*Note:* Data in the same column, the same letter indicates the nonsignificant difference (*p* > 0.05), and different letters indicate the significant difference (*p* < 0.05).

### 3.3 Effect of FLT on Apoptosis in MCF‐7 and MDA‐MB‐231 Cells

**FIGURE 2:**
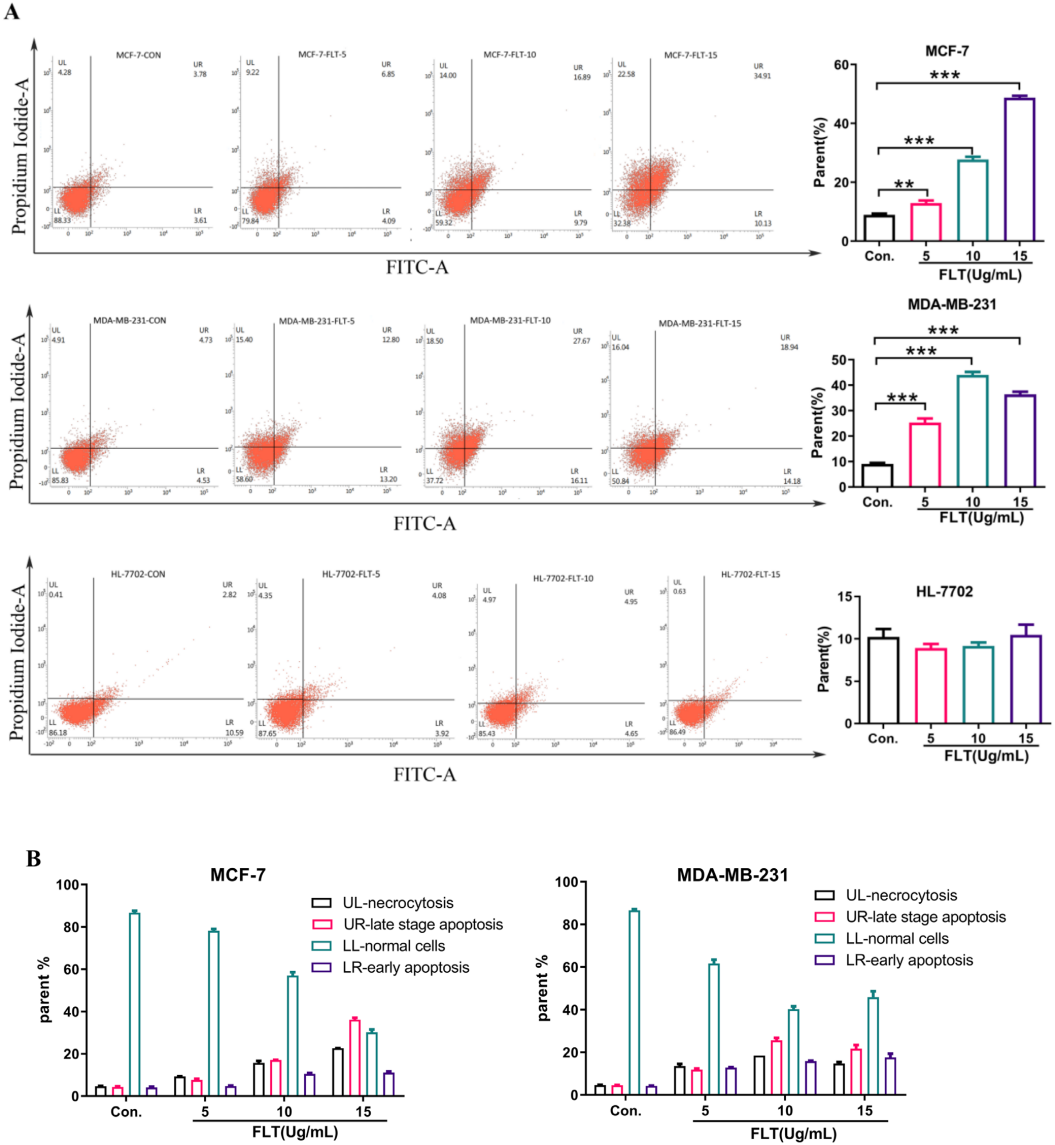
FLT‐induced apoptosis of MCF‐7, MDA‐MB‐231, and HL‐7702 cells. (A) Flow cytometry scatter plots showed the percentage of apoptotic MCF‐7 and MDA‐MB‐231 cells treated with FLT for 24 h. Statistical results showed that FLT induced apoptosis in MCF‐7 and MDA‐MB‐231 cells in a dose‐dependent manner, and the active ingredient FLT induced apoptosis in cancer cells without harming healthy cells. (B) Statistical results showed that FLT induced both early and late apoptosis in MCF‐7 and MDA‐MB‐231 cells in a dose‐dependent manner. Compared with the control group: **p < 0.01, ***p < 0.001.

The induction of apoptosis in cancer cells is a promising approach for cancer treatment (Fulda 2015). Annexin V/PI double staining flow cytometry was used to evaluate the pro‐apoptosis effect of FLT on MCF‐7, MDA‐MB‐231, and HL‐7702 cells. The results demonstrated that compared with the control group, the total apoptosis rate in MCF‐7 and MDA‐MB‐231 cells was significantly increased (*p* < 0.01) after treatment with FLT (5, 10, and 15 μg/mL). Moreover, the proportion of apoptosis increased with the increase in FLT concentration (Figure 2A), indicating that FLT exhibited a concentration‐dependent effect on apoptosis in the two breast cancer cell lines. The total apoptosis rate in HL‐7702 cells treated with the same dose of FLT had no significant change (*p* > 0.05), indicating that the dose of FLT had no toxic effect on HL‐7702 healthy cells.

Phosphatidylserine, a marker of viable apoptotic cells, is specifically bound by Annexin V‐FITC. While nonviable apoptotic cells are simultaneously stained by Annexin V‐FITC and PI, necrotic cells can only be stained by PI. The experimental results showed that FLT exhibited a certain concentration dependence on viable and nonviable apoptosis in MCF‐7 and MDA‐MB‐231 cells; nonviable apoptotic cells accounted for the majority of the total apoptotic cells (Figure 2B). These results indicated that FLT induced apoptosis in MCF‐7 and MDA‐MB‐231 cells.

### 3.4 FLT Uptake and Localization in Cells

**FIGURE 3:**
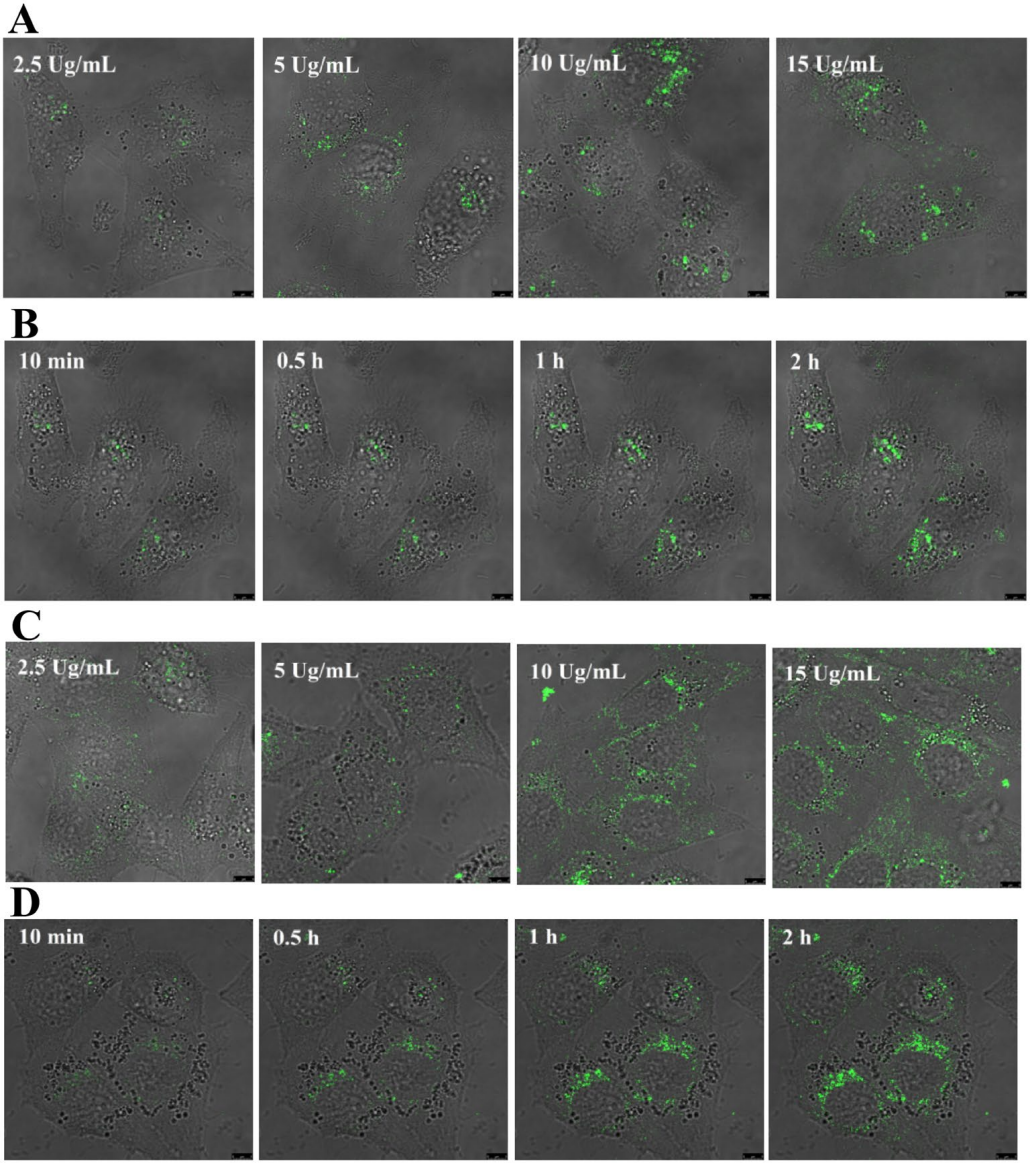
The uptake of FLT in MCF‐7 and MDA‐MB‐231. (A) MCF‐7 cells were treated with different concentrations of FLT for 2 h; (B) MCF‐7 cells were treated by FLT (15 μg/mL) at different times; (C) MDA‐MB‐231 cells were treated with different concentrations of FLT for 2hs; (D) MDA‐MB‐231 cells were treated by FLT (15 μg/mL) at different times.

**FIGURE 4:**
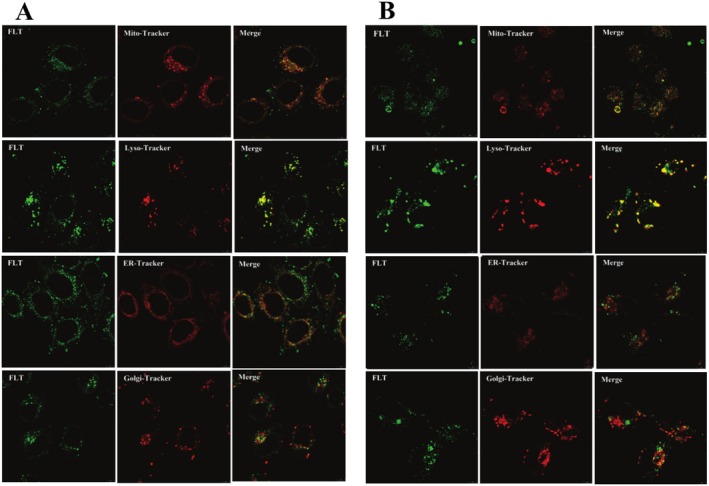
The localization of FLT in MCF‐7 and MDA‐MB‐231. (A) The localization of FLT in MCF‐7. (B) The localization of FLT in MDA‐MB‐231. (The fluorescence intensity of FLT ranged from 89.27–524.60. The fluorescence intensity of mitochondrial marker ranged from 30.92–96.91. The fluorescence intensity of lysosome marker ranged from 38.90–160.36. The fluorescence intensity of ER marker ranged from 32.27–97.59. The fluorescence intensity of Golgi marker ranged from 33.91–58.01.)

Laser confocal imaging is a common method for exploring drug localization in subcellular organelles (Dai et al. 2004). FLT primarily comprises pentacyclic triterpenoids such as UA, OA, and BA, which have similar optical functions. The detection of intracellular fluorescence intensity of FLT revealed bright green fluorescence in water, with the maximum absorption and emission wavelengths at 405 and 500 nm, respectively, and good fluorescence properties. These properties permitted the investigation of the mechanism of its cellular uptake and distribution. Four commercially available organelle probes, Mito‐tracker Red, Lyso‐tracker Red, ER‐tracker Red, and Golgi‐tracker Red, were employed to detect FLT distribution in cells via confocal microscopy. The results revealed that FLT was rapidly absorbed by cells in a dose‐dependent and time‐dependent manner (Figure 3A–D). Moreover, the examination of the combined images indicated that FLT was co‐located with the mitochondrial marker Mito‐tracker Red and the lysosome marker Lyso‐tracker Red but not with Golgi‐tracker Red and ER‐tracker Red (Figure 4A,B), thereby confirming that FLT was located in the mitochondria and lysosome of tumor cells and was less likely to be located in the ER or Golgi apparatus.

### 3.5 Effect of FLT on MMP

**FIGURE 5:**
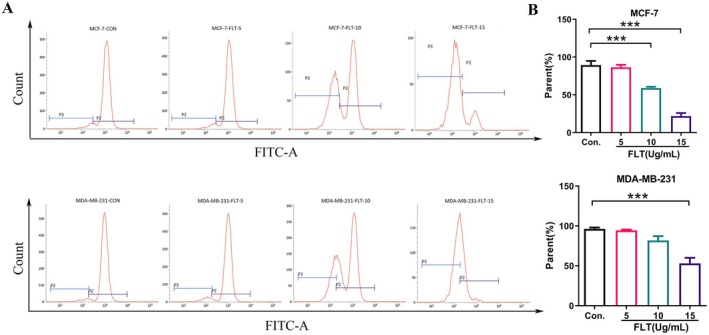
Effect of FLT on MMP. (A) Cells were incubated with FLT at the indicated times, stained with Rh 123, and analyzed by flow cytometry. (B) Statistical results showed that MCF‐7 and MDA‐MB‐231 cells were treated with FLT (5, 10, and 15 μg/mL); MMP decreased in a dose‐dependent manner. Compared with the control group: ***p < 0.001.

Mitochondrial dysfunction can lead to apoptosis in many cancer cells, and MMP loss is a sign of apoptosis (Li et al. 2019; Yu et al. 2022). To investigate whether mitochondria participate in the FLT‐induced apoptosis of MCF‐7 and MDA‐MB‐231 cells, Rh123, a specific fluorescent probe used to analyze MMP, was used for staining and to detect changes in MMP via flow cytometry. The results (Figure 5A,B) showed that compared with the control group, after MCF‐7 and MDA‐MB‐231 cells were treated with FLT (5, 10, and 15 μg/mL), MMP decreased in a dose‐dependent manner. When the concentration of FLT was 15 μg/mL, MMP declined significantly (*p* < 0.001). Thus, combined with the localization results of FLT in cells, the results indicated that FLT might induce apoptosis in MCF‐7 and MDA‐MB‐231 cells through the mitochondrial pathway.

### 3.6 Effect of FLT on Intracellular ROS Levels

**FIGURE 6:**
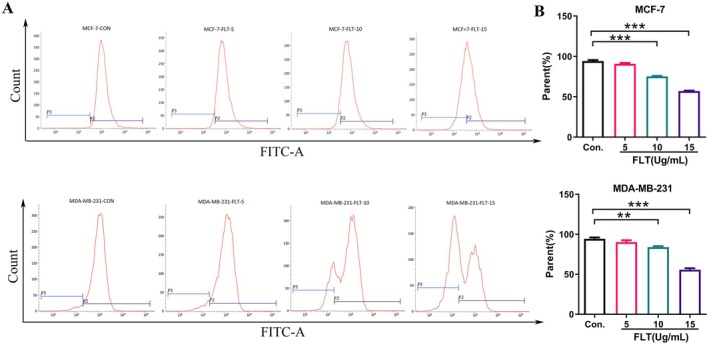
Effect of FLT on intracellular ROS level. (A) Cells were incubated with FLT at the indicated times, stained with DCFH‐DA, and analyzed by flow cytometry. (B) Statistical results showed that MCF‐7 and MDA‐MB‐231 cells were treated with FLT (5, 10, and 15 μg/mL); the intracellular ROS level decreased in a dose‐dependent manner. Compared with the control group: *p < 0.05, **p < 0.01, ***p < 0.001.

Mitochondria are the main source of intracellular ROS. To detect ROS levels in cells, DCFH‐DA staining and flow cytometry were performed. The results (Figure 6A,B) showed that compared with the control group, the intracellular ROS levels in MCF‐7 and MDA‐MB‐231 cells treated with FLT (5, 10, and 15 μg/mL) decreased in a dose‐dependent manner. When the concentration of FLT was 15 μg/mL, ROS levels declined significantly (*p* < 0.001), indicating that FLT may induce apoptosis in MCF‐7 and MDA‐MB‐231 cells by reducing the intracellular ROS levels.

### 3.7 Effect of FLT on Apoptosis‐Related Protein Level

**FIGURE 7:**
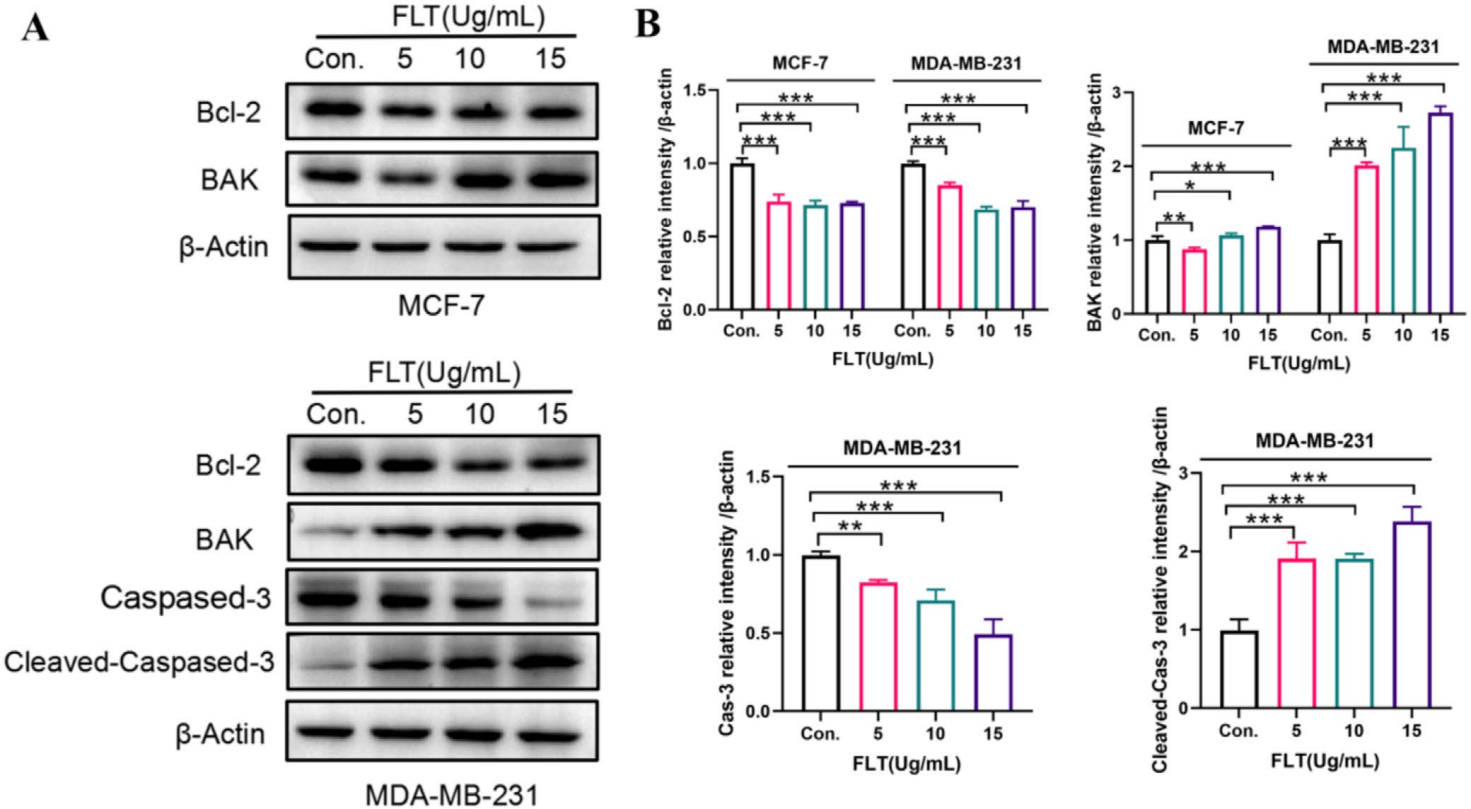
Effect of FLT on apoptosis‐related protein level. (A) Western blot. (B) Relative expression level of proteins. Compared with the control group: *p < 0.05, **p < 0.01, ***p < 0.001.

The Bcl‐2 family plays a central role in the occurrence of apoptosis mediated by the mitochondrial pathway. The Bcl‐2 family members include the pro‐apoptotic protein Bak/Bax and anti‐apoptotic protein Bcl‐2 (Bcl‐2, B‐cell lymphoma‐extra large (Bcl‐XL), BCL‐2 like 2 (Bcl‐W), and myeloid cell leukemia 1 protein (MCL‐1)) (Youle and Strasser 2008; Liu et al. 2021). Bak usually exists on the outer membrane of mitochondria in the form of an inactive monomer in cells as well as a key protein in cell apoptosis. Apoptosis signals in cells quickly activate Bak, forming holes on the outer membrane of mitochondria, releasing the second messenger that expands apoptosis signals, and promoting cell death. Meanwhile, the ratio of the pro‐apoptotic protein to anti‐apoptotic protein determines the survival or death of injured cells. An increased ratio leads to MMP reduction and cytochrome C release, thereby activating the downstream caspase family. Caspase‐3 is the main effector molecule in the apoptosis process, and its activation indicates the irreversible stage of apoptosis (Singh and Kumar 2016; Liu et al. 2020). The present study results (Figure 7A,B) demonstrated that the Bcl‐2 level was significantly reduced (*p* < 0.001), whereas the Bak level was significantly increased (*p* < 0.05) after the FLT (5, 10, and 15 μg/mL) treatment of MCF‐7 and MDA‐MB‐231 cells for 24 h; caspase‐3 level was significantly reduced (*p* < 0.01), whereas cleaved‐caspase‐3 level was significantly increased (*p* < 0.001) after the FLT (5, 10, and 15 μg/mL) treatment of MDA‐MB‐231 cells for 24 h. Compared with the control group, FLT could regulate the expression of Bcl‐2, Bak, caspase‐3, and cleaved‐caspase‐3 in a dose‐dependent manner. Thus, these results and the localization of FLT in cells and its effect on MMP indicated that FLT induced apoptosis in MCF‐7 and DA‐MB‐231 cells through the mitochondrial pathway.

### 3.8 Effect of FLT on Mitochondrial Division‐Related Protein Level

**FIGURE 8:**
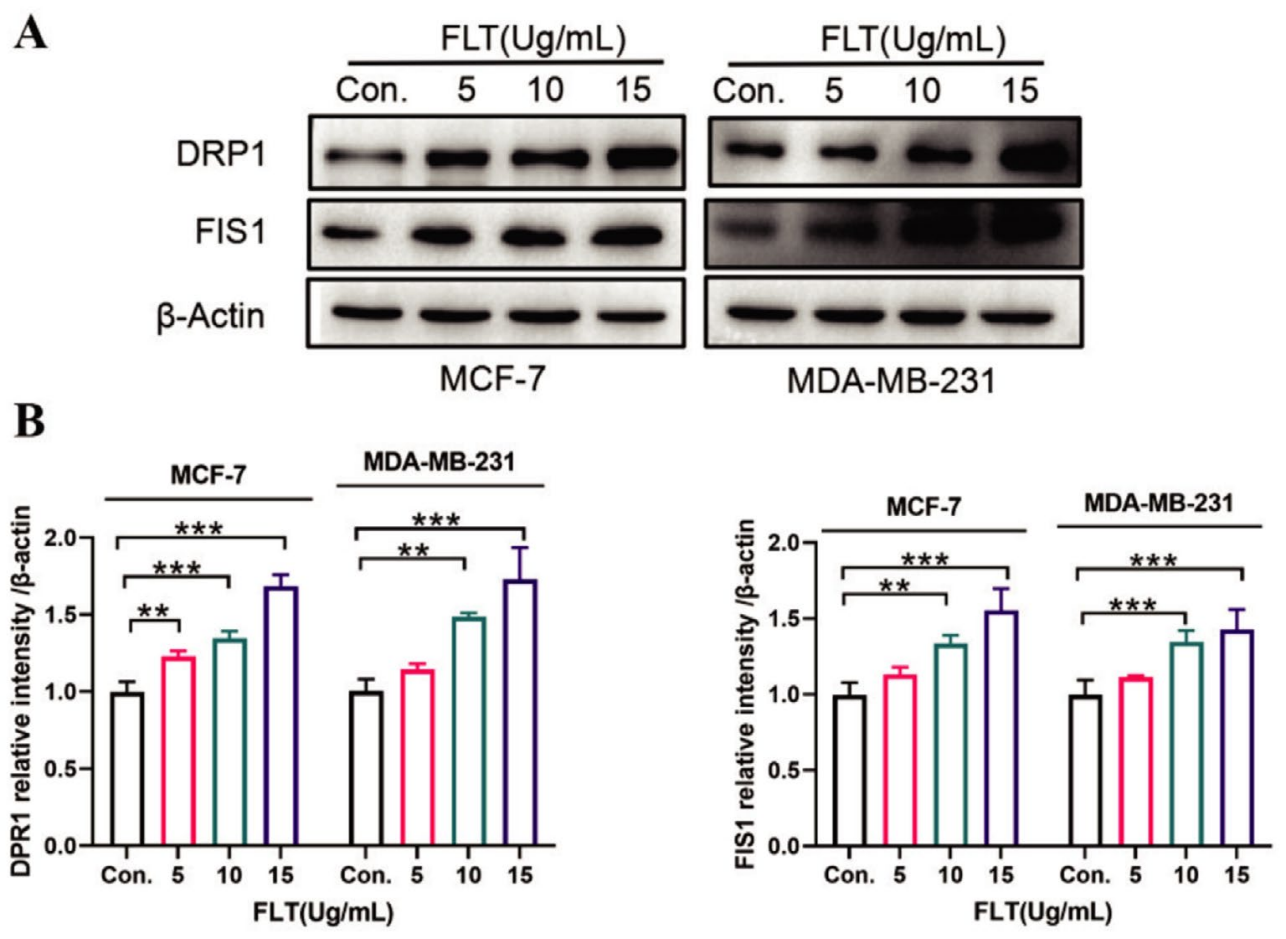
Effect of FLT on mitochondrial division‐related protein level. (A) Western blot. (B) Relative expression level of proteins. Compared with the control group: **p < 0.01, ***p < 0.001.

DRP1 is the main executor of mitochondrial division in mammals and an important target for tumor initiation, migration, proliferation, and chemosensitivity (Katajisto et al. 2015; Xie et al. 2015). Under normal circumstances, DRP1 is present in the cytoplasm. During mitochondrion division, DRP1 translocates to the surface of mitochondria and combines with mitochondrial receptor proteins such as FIS1 on the outer membrane of mitochondria to form a circular structure that can narrow mitochondria and induce mitochondrial division (Hu et al. 2017; Zhang, Jia, et al. 2016; Zhang, Liu, et al. 2016). The current study results (Figure 8A,B) showed that after the FLT (10 and 15 μg/mL) treatment of MCF‐7 and MDA‐MB‐231 cells for 24 h, DRP1 and FIS1 levels were significantly increased (*p* < 0.01) and that, compared with the control group, FLT induced DRP1 and FIS1 production in a dose‐dependent manner. These results indicated that FLT may induce apoptosis in MCF‐7 and MDA‐MB‐231 cells by promoting DRP1 and FIS1‐mediated mitochondrial division.

## 4 Discussion

The fruit of *F. suspensa* is a kind of Chinese herbal medicine commonly used in China. In Chinese medicine, *F. suspensa* is used to prepare formulas with other herbs for the treatment of head and neck tumors, breast cancer, esophageal cancer, gastric cancer, cervical cancer, leukemia, and other tumors. The extract of *F. suspensa* fruit has shown better antitumor effects in animal experiments and in vitro studies; alcoholic fruit extracts have been shown to exhibit better effects than aqueous extracts (Wei et al. 2020; Yan et al. 2012; Sun et al. 2015; Zhao et al. 2015). However, the antitumor activity of *F. suspensa* leaves has not been reported. In Beitai, Aihaoping, and Mapaoquan villages in Wuding City, Hebei Province, people have consumed *F. suspensa* leaf tea since antiquity. Of note, these villagers seldom suffer from cancer, cerebral stagnation, and other diseases. Therefore, these villages are known as “longevity villages.” It is assumed that drinking *F. suspensa* leaf tea is associated with the prevention of tumors and cardiovascular diseases. Thus, *F. suspensa* leaves may be used as an antitumor plant‐based source with the prospect of further development and utilization for anticancer treatment.

Preliminary screening has revealed that FLT may be the main antitumor active ingredient of the leaves (Wang 2022). In the current study, the total FLT was extracted and concentrated from *F. suspensa* leaves. HPLC revealed UA, OA, and BA as the primary components, whose total level reached 91.49%. MTT assay was performed to analyze cell proliferation, Annexin V‐FITC/PI double staining and flow cytometry were used to detect cell apoptosis, and laser confocal microscopy was employed to detect the uptake and location of FLT in cells. Moreover, the Rh123 staining method was used to detect changes in MMP, and the DCFH‐DA staining method was employed to detect changes in ROS level in cells. Finally, western blotting was employed to detect the expression of apoptosis‐related protein caspase‐3, cleaved‐caspase‐3, Bcl‐2, Bak, DRP1, and FIS1 to explore the effect of FLT on apoptosis in human breast cancer cells MCF‐7 and MBA‐MB‐231 along with the mechanism.

In the antiproliferation experiment in vitro, FLT, UA, and OA exhibited strong inhibitory effects on the cell lines, particularly on human breast cancer cell lines. Among the three, FLT had the strongest antiproliferative activity and its IC_50_ values of antiproliferative activity on human breast cancer cell lines (MCF‐7 and MDA‐MB‐231) were (6.52 ± 0.79) μg/mL and (7.90 ± 0.94) μg/mL, respectively. In addition, Annexin V‐FITC/PI double staining flow cytometry showed that FLT could induce apoptosis in MCF‐7 and MDA‐MB‐231 cells.

Studies have shown that the occurrence and development of tumors are correlated to apoptosis and that mitochondria have a regulatory effect on apoptosis (Gogvadze et al. 2008; Zhu and Chen 2008). Apoptosis can be initiated via three mechanisms: mitochondrial pathway, death receptor pathway, and endoplasmic reticulum pathway. Of these, the mitochondrial pathway plays an important role in mediating apoptosis (Nichani et al. 2020; Zhang et al. 2022). During cell apoptosis, the integrity of the mitochondrial membrane is destroyed, with MMP decline a sign of early cell death (Xu et al. 2021). In the present study, Rh123 staining and flow cytometry detection results showed that FLT could significantly reduce the MMP of MCF‐7 and MDA‐MB‐231 cells. Similarly, Liu et al. (2023) reported that the *Ganoderma lucidum* triterpenoid compound Lucialdehyde B can significantly reduce the mitochondrial membrane potential of nasopharyngeal carcinoma CNE2 cells and induce cell apoptosis. Moreover, the uptake and localization of FLT in cells were detected using laser confocal microscopy, and the results showed that FLT was located in the mitochondria, lysosome, and not in the ER or Golgi apparatus. These results suggested that FLT induces apoptosis in tumor cells via the mitochondrial pathway.

Mitochondria are the main production site of ROS. As the second‐most important intracellular messenger, ROS can trigger several key signaling pathways, including tumor cell proliferation, apoptosis, metastasis, and tumor angiogenesis, and ultimately lead to the development of malignant tumors. Many studies have reported that cancer cell apoptosis is accompanied by an increase in ROS production (Ko et al. 2016; Li et al. 2017; Zhang, Jia, et al. 2016; Zhang, Liu, et al. 2016) and that certain compounds can induce apoptosis in cancer cells while reducing ROS production (Liu et al. 2014; Ma et al. 2019). Therefore, there is a delicate balance in ROS production. Too much or insufficient ROS may cause cancer cell death (Trachootham et al. 2009). The present study revealed that FLT can significantly reduce ROS levels in breast cancer cells in a dose‐dependent manner while inducing apoptosis in breast cancer cells. Although the mechanism of FLT interfering with ROS production and subsequently leading to apoptosis needs further investigation, these results suggest that the mitochondria‐dependent apoptosis pathway is involved.

The mitochondrial apoptosis pathway exerts its role mainly through the action of the upstream signaling molecules of mitochondria on the mitochondrial membrane after activation, leading to changes in the functional state of the mitochondrial membrane and activation of the Bcl‐2 protein family. When the mitochondria permeability transition pore opens, the apoptotic active substance cytochrome C, which is involved in electron transmission between the inner and outer membranes of mitochondria, is released into the cytoplasm to activate the caspase cascade reaction, introduce breaks in DNA, and produce apoptotic bodies, thereby promoting apoptosis (Qian et al. 2023; Xia et al. 2023). The current study discovered that after the FLT treatment of MCF‐7 and MDA‐MB‐231 cells for 24 h, the Bcl‐2 level decreased and Bak, activated caspase‐3, and cleaved‐caspase‐3 levels increased, indicating that FLT induced apoptosis in MCF‐7 and MDA‐MB‐231 cells through the mitochondrial pathway. The study by Chu et al. (2021) also reported similar results. When investigating the anticancer activity of oleanolane triterpenes extracted from Nansheteng ( *Celastrus orbiculatus* ) in the gastric cancer BGC‐823 cell line, it was found that it could significantly reduce the mitochondrial membrane potential of BGC‐823 cells, induce cell apoptosis, and at the same time, down regulate Bcl‐2 protein expression and up regulate cleaved‐caspase‐3 protein expression. In addition, Cheng et al. (2018) showed that the triterpene compound *Clematis* hederagenin saponin can significantly reduce the level of cytochrome C protein expression in breast cancer cells, up regulate caspase‐3 enzyme activity, and induce programmed death of breast cancer cells.

In addition to being the energy source of cells, mitochondria perform the functions of regulating cellular immunity, calcium homeostasis, and autophagy, and play a role in adjusting the dynamic balance of division/fusion of their morphology (Tilokani et al. 2018). Indeed, changes in their dynamic state can affect the occurrence, development, and metastasis of tumors (Zemirli et al. 2018). Research has shown that increased mitochondrial division can cause apoptosis in breast cancer cells (Tang et al. 2018). Some scholars believe that mitochondrial division is an essential step in cell apoptosis, in which DRP1 recruitment in mitochondria plays a key role. The activation of Bax/Bak leads to the colocation of Bax and DRP1 at the mitochondrion site where division occurs. DRP1 stably binds to the outer membrane of mitochondria, leading to mitochondrial fragmentation (Karbowski et al. 2002; Wasiak et al. 2007). The results of the present study revealed that after the FLT treatment of MCF‐7 and MDA‐MB‐231 cells for 24 h, the expression of DRP1 and FIS1 increased, indicating that FLT induced apoptosis in MCF‐7 and MDA‐MB‐231 cells by promoting mitochondrial fission mediated by DRP1 and FIS1. The results also suggested that FLT‐induced apoptosis in breast cancer cells may be related to changes in mitochondrial division.

## 5 Conclusion

Taken together, the present study results confirmed that FLT has a good antiproliferative activity against breast cancer cells. FLT promoted apoptosis in MCF‐7 and MDA‐MB‐231 cells through the mitochondrial pathway. The possible mechanism is that FLT affects the expression of the mitochondrial Bcl‐2 family by promoting mitochondrial division mediated by DRP1 and FIS1, thereby activating caspase‐3 and ultimately causing apoptosis. Therefore, *F. suspensa* leaves may be used as an antitumor plant‐based raw material with great prospects for development and utilization for anticancer treatment. Although this study suggests that FLT may promote apoptosis through DRP1 or FIS1 induced mitochondrial division and fusion, its mechanism of action still needs further investigation, such as through siRNA‐mediated DRP1 or FIS1 inhibition experiments to further verify their roles in mitochondrial division and apoptosis.

## Author Contributions

**Xiao Li:** conceptualization (equal), data curation (equal), formal analysis (equal), investigation (equal), resources (equal), writing – original draft (equal). **Xuefang Wang:** conceptualization (equal), data curation (equal), writing – review and editing (equal). **Ling Chen:** conceptualization (equal), data curation (equal), formal analysis (equal), supervision (equal). **Erjuan Ning:** methodology (equal), resources (equal), writing – review and editing (equal). **Hang Fan:** investigation (equal), methodology (equal). **Jun Liang:** investigation (equal), project administration (equal). **Xinxin Lai:** investigation (equal), software (equal). **Lulu Zhang:** supervision (equal), visualization (equal). **Yingkui Gao:** investigation (equal), resources (equal). **Yan Li:** software (equal), validation (equal). **Panpeng Wei:** formal analysis (equal), investigation (equal). **Liqin Yu:** resources (equal). **Yi Fan:** conceptualization (equal), formal analysis (equal), funding acquisition (equal), project administration (equal), resources (equal). **Xuebing Wang:** conceptualization (equal), formal analysis (equal), funding acquisition (equal), resources (equal).

## Conflicts of Interest

The authors declare no conflicts of interest.

## Data Availability

The data that support the findings of this study are available from the corresponding author upon reasonable request.
